# How To Build an Integrated Neighborhood Approach to Support Community-Dwelling Older People?

**DOI:** 10.5334/ijic.1596

**Published:** 2016-05-12

**Authors:** Hanna Maria van Dijk, Jane Murray Cramm, Anna Petra Nieboer

**Affiliations:** Institute of Health Policy and Management, Erasmus University Rotterdam, the Netherlands; Associate Professor, Institute of Health Policy and Management, Erasmus University Rotterdam, the Netherlands; Professor, Institute of Health Policy and Management, Erasmus University Rotterdam, the Netherlands

**Keywords:** integrated care and support, integrated neighborhood approach, community level, community-dwelling older people, informal support, the Netherlands

## Abstract

**Background:**

Although the need for integrated neighborhood approaches (INAs) is widely recognized, we lack insight into strategies like INA. We describe diverse Dutch INA partners’ experiences to provide integrated person- and population-centered support to community-dwelling older people using an adapted version of Valentijn and colleagues’ integrated care model. Our main objective was to explore the experiences with INA participation. We sought to increase our understanding of the challenges facing these partners and identify factors facilitating and inhibiting integration within and among multiple levels.

**Methods:**

Twenty-one interviews with INA partners (including local health and social care organizations, older people, municipal officers, and a health insurer) were conducted and subjected to latent content analysis.

**Results:**

This study showed that integrated care and support provision through an INA is a complex, dynamic process requiring multilevel alignment of activities. The INA achieved integration at the personal, service, and professional levels only occasionally. Micro-level bottom-up initiatives were not aligned with top-down incentives, forcing community workers to establish integration *despite* rather than *because of* meso- and macro-level contexts.

**Conclusions:**

Top-down incentives should be better aligned with bottom-up initiatives. This study further demonstrated the importance of community-level engagement in integrated care and support provision.

## Background

Many Western countries face the challenge of meeting the needs of increasing numbers of care-dependent older people using limited health and social care budgets. The development of sustainable long-term care systems that adequately address these needs strains countries’ innovative capacities forcing nations to restructure the division of responsibilities among the state, market, and community [1,2]. Instead of the state serving as the main provider of (social) care, such burdens have been allocated to communities in many Western countries [3,4,5,6]. In this framework, public protection is provided only when the community cannot provide care for objective reasons, such as the absence of informal caregivers and/or insufficient economic means [2]. As increasing numbers of older people continue to live at home, an integrated neighborhood approach (INA) is needed [7]. Integrated care approaches need to incorporate the recognition that communities are co-producers of health and well-being [8,9,10]. INAs, consisting of collaboration among municipalities, health and social care, informal care providers, voluntary/third sector, churches, schools and the private sector are therefore increasingly advocated as means to overcome current service fragmentation and co-ordinate care and support according to people’s (complex) needs [11,12,13]. INAs aim is to use available neighborhood resources effectively and increase responsiveness to citizens’ specific needs, ensuring the provision of person- and population-centered support [13,14]. Person-centered care and co-ordination among both formal and informal care are now widely recognized as a critical component of privately and publicly funded health care despite significantly different systems of care [15].

### An INA in Rotterdam

INA was supported by a grant provided by the Netherlands Organization for Health Research and Development (ZonMw) as part of the National Care for the Elderly Program, which was designed to improve care for elderly people with complex care needs throughout the Netherlands. The National Care for the Elderly program started in April 2008 and will run until 2016. Funds for INA were also received from the Dutch Healthcare Authority (NZa), Geriatric Network Rotterdam and the municipality of Rotterdam. In 2011, the Rotterdam municipality, local health and social care organizations, Erasmus University Rotterdam, the University of Applied Sciences, and Geriatric Network Rotterdam initiated the INA for community-dwelling older people in Rotterdam called ‘Let’s Talk’ (Even Buurten) in which the municipality took the lead [16]. Its overarching aim was to create a supportive environment allowing community-dwelling older people to live independently. Although health and social care services are widely available in Rotterdam, they are often fragmented and lack outreach activities that foster early identification of frail older people. The need to invest in (preventive) strategies facilitating older people’s ability to continue living at home has increased with municipal legal responsibilities related to social services (e.g. home care and support of older people and informal support-givers). To achieve this goal the INA needs to overcome barriers associated with the provision of care and support for older people in the Netherlands, reinforce networks among health and social care providers and informal support-givers in the community, based on recognition of their mutual dependence in efforts to optimize current services [16,17].

**Figure 1:**
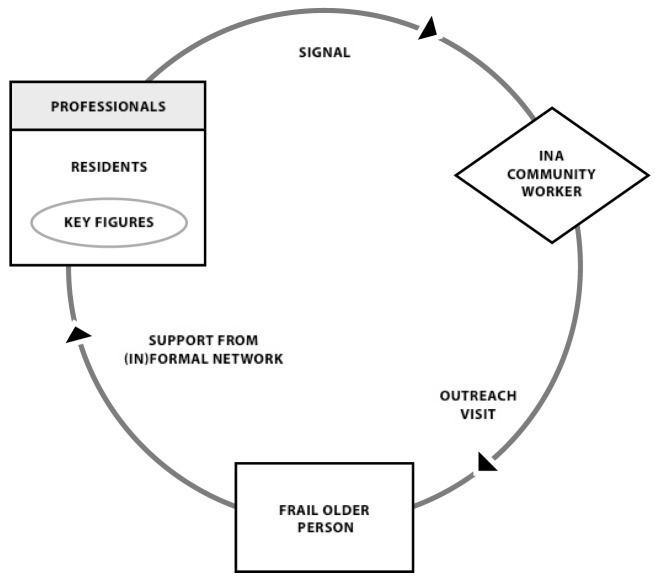
Working method INA.

The INA’s success in providing person- and population-centered care and support requires collaboration among formal and informal community partners on aspects of care ranging from the signaling of problems to prevention and support. Within the INA context, professionals and residents were asked to watch over neighbors and report manifestations of frailty among older people to INA community workers (Fig. 1). Early detection and case finding is crucial to support older people to age in place. Residents often notice changes and deteriorations in older people’s lives at an earlier stage than professionals. Key figures who are the ‘eyes and ears’ of the neighborhoods are represented by active residents as well as professionals working in these areas (e.g. general practitioners, social workers, police officers). If key figures notice an older person might be in need of support they are expected to reach out to the community worker of the INA via a signal. These community workers have health and social care backgrounds and have been temporarily reassigned to INA teams, which often include at least one social worker and one community nurse familiar with the neighborhood. Community workers visit older people at home and map their wishes and needs *via* phased interviews. In consultation with older people, community workers seek appropriate solutions within (preferably informal) networks. The project’s study protocol [17] contains more information on its scope and aims.

### Theoretical framework

**Figure 2:**
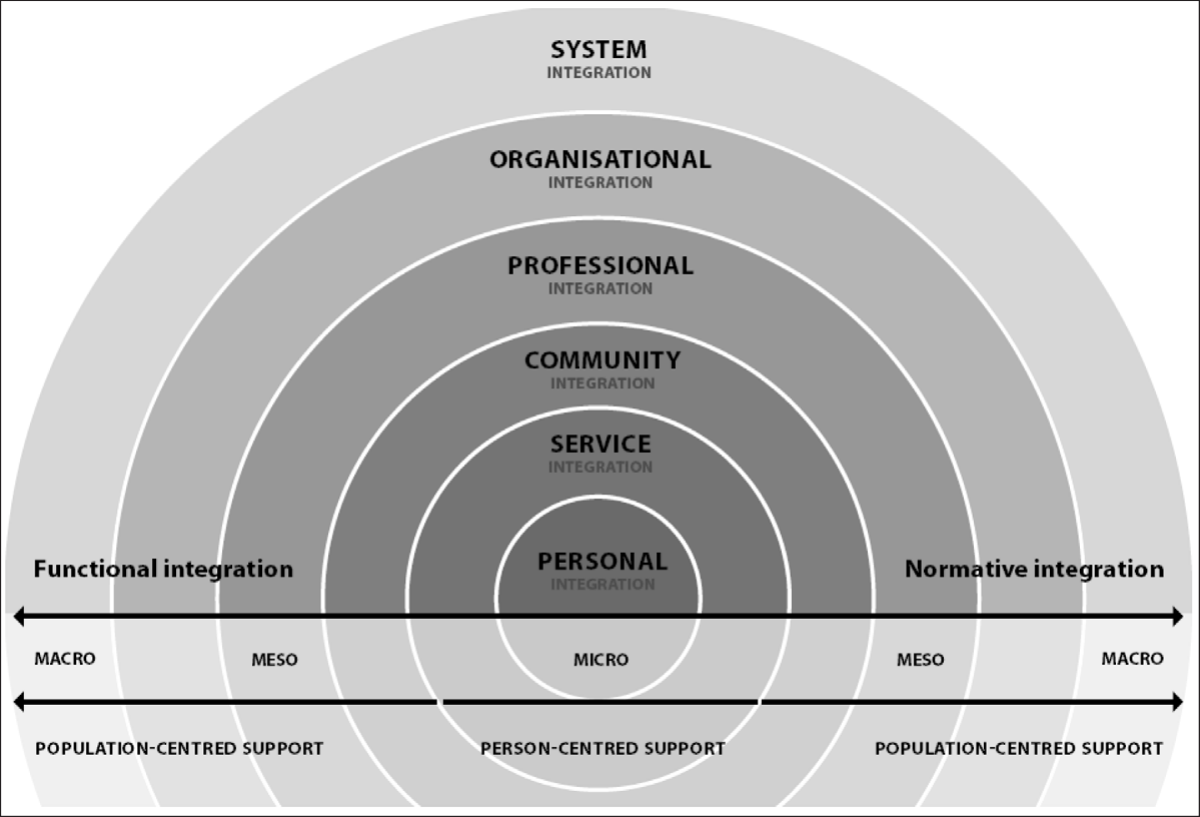
Integrated care model van Dijk, Cramm and Nieboer (adapted from Valentijn et al., 2013).

As INAs depend on stakeholders’ continuous involvement and interdependence across multiple levels, gaining insight into factors that hinder or facilitate community-based integrated care and support is important. INA’s success depends on integration within and among the micro-level (primary delivery of care and support), meso-level (community, professional, organizational contexts), macro-level (broader policy context of care and support systems), functional integration, and normative integration (Fig. 2) [13,18,19].

At the micro-level, personal integration involves a holistic and coordinated approach to an older person’s health and well-being, requiring professionals’ active engagement and support of his/her self-management abilities [18]. Service integration ensures the provision of tailored and coherent services across time, space, and disciplines [20]. To support a person-centered, rather than disease-oriented, approach, assessment tools and instruments should account for overall well-being [19]. Micro-level integration thus requires collective actions of partners across the entire care and support continuum.

The meso-level encompasses structures that exceed community, professional, and organizational boundaries [13]. Integrated care and support models often neglect the community level, which is crucial in increasing responsiveness to older people’s needs and bundling resources available among formal and informal support-givers [13]. Therefore, we added community integration to the meso-level in the adapted model. On a professional level, partnerships within and among health and social care organizations are needed. These partnerships ideally cover a range of specialist and generalist skills to enable a holistic approach to older peoples’ needs. Organizational integration aims to overcome organizational boundaries that may hamper collaboration among health and social care professionals. It provides structural activities that promote collaboration among organizations [18,19].

On the macro-level, the system should account for the complexity of issues that arise locally with respect to person- and population-centered support provision. It should thus provide regulatory, accountability, and financial incentives that stimulate integrated care and support realization on the meso- and micro-levels [19].

Integration should also focus on the multilevel alignment of activities [18,19]. Functional integration focuses on the coordination of support functions, such as information management, skilled leadership, and quality improvement. Normative integration is a less tangible, yet essential, dimension involving the creation of an integrated mind-set and common set of values [21].

### Aims

Although the need for INAs to achieve a better balance between supporting increasing numbers of care-dependent older people and reducing public spending is widely recognized, we lack insight into strategies like INA. In this case study of an INA in Rotterdam, the Netherlands, we describe collaboration among diverse community partners to provide person- and population-centered integrated care and support to community-dwelling older people using an adapted version of Valentijn and colleagues’ [19] integrated care model. Our main objective was to explore the experiences of municipal officers, health insurers, health and social care organizations, and older people with INA participation. We sought to increase our understanding of the challenges facing these partners and identify factors facilitating and inhibiting integration within and among multiple levels.

## Methods

### Design and setting

This qualitative, descriptive study was based on face-to-face interviews with INA participants conducted in several districts of Rotterdam over a 4-month period in 2013. The first author also made field notes and audio-recordings at several INA-related meetings, ranging from those of community-based teams and civic steering committees to educational meetings for community workers. The INA was initiated in two districts of Rotterdam in April 2011 and extended to two additional districts 1 year later. The ethics committee of Erasmus University Medical Centre of Rotterdam approved the project in June 2011 (MEC-2011-197).

### Sample

The sample consisted of 21 INA participants, including the INA project manager, three older people who received INA support, four INA community workers with health and social care backgrounds, four managers/directors of health and social care organizations, seven municipal officers, one health insurer, and one former politician who remained actively engaged in the field of long-term care (see Table 1 for more details). Professionals were selected on the basis of their participation within INA, ensuring a variety of participants in terms of (professional) background (e.g. health or social care background) and responsibilities (e.g. on an executive, managerial or policy level). In addition to our own selection of participants that we deemed essential to interview, we used snowball sampling to identify new participants that could contribute to our study. Last, we relied on INA’s community workers to identify and invite older people who received INA support to participate in the study.

**Table 1:** Study participants.

Participant	Gender	Background

***Community workers***		
*Participant 1*	woman	Community nurse INA with a social care background (specialized in coordinating voluntary work)
*Participant 2*	woman	Community nurse INA with a health care background (specialized as a nurse practitioner)
*Participant 3*	man	Community nurse INA with a social care background (specialized in community work)
*Participant 4*	woman	Community nurse INA with a social care background (specialized in community work)
***Managers/directors***		
*Participant 5*	man	Manager health care organization
*Participant 6*	man	Manager social care organization
*Participant 7*	woman	Manager health care organization
*Participant 8*	woman	Director health care organization
***Municipal officers***		
*Participant 9*	woman	Alderman (with a portfolio responsibility on participation and integration)
*Participant 10*	woman	Alderman sub-municipality (with a portfolio responsibility on health and social care)
*Participant 11*	man	Senior policy officer Social Support Act
*Participant 12*	man	Program manager assisted living
*Participant 13*	woman	Policy officer sub-municipality health and social care
*Participant 14*	woman	Policy officer sub-municipality health and social care
*Participant 15*	man	Policy officer health and social care
***Older people***		
*Participant 16*	woman	Older person who received INA support and resided in Oude Westen
*Participant 17*	woman	Older person who received INA support and resided in Lombardijen
*Participant 18*	woman	Older person who received INA support and resided in Kralingen
***Other***		
*Participant 19*	man	Project manager of INA
*Participant 20*	man	Director procurement and policy of a health insurance company
*Participant 21*	woman	Former politician who remained actively engaged in the field of long-term care (e.g. through her participation as a program member of the National Care for the Elderly Program)

### Interviews

The first author conducted all interviews (60–90 minutes) at participants’ offices or homes; one interview involved three municipal officers simultaneously. Interviews were audio-taped with participants’ permission and transcribed. The interviews aimed to elicit participants’ reflections on their experiences with the INA from their (professional) perspectives. Because relevant research is sparse, performing interviews with a limited number of preconceived categories was most appropriate [22]. To gain new insight, we allowed themes to arise from the data [22,23]. Participants were encouraged to describe and reflect on their experiences with (collaborative or competitive) interaction among community partners and the perceived roles and responsibilities with respect to integrated care and support provision to older people. More specifically, older people were asked to describe their experiences with INA; i.e. how they got involved with INA, their initial expectations of INA, their perspectives on INA’s core principles (e.g. with respect to informal support-giving and encouragement of self-management abilities) and their overall experiences with INA support-giving. Next, the interviews with INA community workers and project manager aimed to elicit their roles and responsibilities within INA, their experiences with successful/difficult parts of their work, their perspectives on INA’s core principles and their views on micro-, meso- and macro-level factors that impeded or facilitated these principles. Last, the goal of the interviews with the other participants (i.e. managers/directors of health and social care organizations, municipal officers, the health insurer and former politician) was to gain an overview of the different micro-, meso- and macro-level facilitators and barriers they faced when dealing with integrated care and support-giving to community-dwelling older people in general as well as with INA in particular.

### Analysis

Latent content analysis of narrative text was performed, which yields a rich understanding of a phenomenon [22,24]. To avoid loss of nuance, participants’ narratives were translated into English only in the report-writing stage. To obtain a holistic perspective, entire transcribed texts were first read open-mindedly several times. Transcriptions from each group were then read separately to comprehend overall meaning. Then, we read texts word by word, extracting ‘units of meaning’ that were coded and categorized using atlas.ti. Finally, the underlying meanings (i.e. latent content) of categories were formulated into themes [24].

Barriers to and facilitators of integration were identified within our theoretical framework of the adapted integrated care model [19]. Results are reported by integration level, with quotations identified by participants’ backgrounds [community worker (CW), older person (OP), project manager (PM), health or social care director/manager (HCD/SCD/HCM), sub-alderman (SUBALD), municipal officer (MO), health insurer (HI), and head education team (HET)]. Elements contributing to functional and normative integration among levels were also examined.

## Results

Table 2 presents the main findings of our study.

**Table 2:** Overview of our study findings.

Integration level	Challenge	Key observations

***Micro-level***		
*Personal integration*	Gaining trust	Obtaining older people’s trust was identified as a key prerequisite for the provision of person-centered support. Continuity, in turn, is a precondition for gaining trust.
*Personal integration*	Acknowledging and strengthening older people’s capabilities	The INA uses individualized support plans based on assessments of older people’s physical and social needs *and* capabilities.
*Personal integration*	Overcoming resistance to informal support	Community workers reported that older people had difficulty relying on informal networks; they were reluctant to ask for help and strongly desired independence.
*Service integration*	Engaging community resources	Community workers tried to mobilize volunteers to set up services, which was not always successful.
*Service integration*	Community workers must set up *and* track responses to interventions	To ensure service integration, community resources must be integrated throughout the process of signaling and supporting older people. Moreover, integrated care and support provision requires community workers to operate simultaneously at multiple levels.
***Meso-level***		
*Community integration*	Building community awareness and trust	Community workers noted that conveying the INA’s message took time and that community members often hesitated to alert them to frail older persons, reluctant to interfere in someone’s life.
*Community integration*	Familiarity with the neighborhood	INA community workers must take the preferences, and sometimes prejudices, of support-givers and those in need of support into account.
*Community integration*	Adaption to new roles	The need for community integration requires professionals to reinvent their roles and serve as community workers.
*Community integration*	Sustaining relationships	To overcome barriers to community integration, community workers perceived that sustaining relationships was crucial in gaining access to frail older people and adequately assessing potential support-givers.
*Professional integration*	Individual skills	Recruitment of ‘entrepreneurial’ professionals with generalist and specialist skills to form diverse teams was crucial for professional integration.
*Professional integration*	Team skills	Discontinuity and a lack of mutual goals were found to hamper professional integration.
*Organizational integration*	Conflicting organizational interests	Although health and social care organizations recognize the need to collaborate, professionals feel that cost containments are forcing the prioritization of organizations’ interests over the common good.
*Organizational integration*	Lack of organizational commitment	Organizational integration was impeded by conflicting organizational interests and achieved only under favorable conditions, i.e. through a few willing professionals or managers and through high levels of trust built during previous collaborations. Structural incentives, such as the creation of opportunities for professionals to meet and gain insight in each other’s added value, facilitate organizational integration.
***Macro-level***		
*System integration*	Inadequate financial incentives	Participants identified divergent flows of funds as the main cause for the lack of adequate financial incentives, affecting health and social care organizations and municipalities.
*System integration*	Inadequate accountability incentives	Health and social care organizations urged the municipality to reconsider its accountability incentives, annoyed by the focus on *how* they do things.
*System integration*	Inadequate regulatory incentives	Community workers are told that the provision of high-quality support requires innovation and collaboration among community partners while being required to bureaucratically account for all actions and meet targets.
***Functional integration throughout all levels***	The risk of excessive professional autonomy	Professional autonomy provided by project management was at odds with guidance in adopting a new professional role that matched the INA’s core principles.
	Lack of support tools	The INA’s innovative character increased community workers’ need for guidance and supportive tools. The lack of material (i.e. decision-support tools or guidelines) and immaterial (i.e. acknowledgement) resources hampered the creation of shared values and aligned professional standards.
	High touch, low tech	In exchanging information, community workers often applied a ‘high touch, low tech’ approach. Rather than using the web-based portal developed for the INA, community workers preferred to consult each other by telephone or in person.
***Normative integration throughout all levels***	Insecurity and mistrust	For older people, tender practices and policy changes often implied the rationing of publicly funded health and social care services and discontinuity in service delivery. Municipalities were similarly affected by a high degree of insecurity.

### Micro-level: personal integration

#### Gaining trust

Obtaining older people’s trust was identified as a key prerequisite for the provision of person-centered support: *“older people are very suspicious. And from that distrust they need trust, someone they can trust”. (HCD)*

Continuity is a precondition for gaining trust. Rapid fluctuations in projects often have resulted in discontinuities in care and support co-ordination, rendering older people distrustful of new projects and faces. Their awareness of their frail condition exacerbates this distrust. INA community workers thus had to invest much time in becoming familiar faces in neighborhoods. The use of business cards and posters with their photographs contributed to their familiarity, and older people reported that they kept these business cards at hand. Older people who built relationships with community workers felt reassured that they could confide in them and rely on them when in need.

#### Acknowledging and strengthening older people’s capabilities

The INA uses individualized support plans based on assessments of older people’s physical and social needs *and* capabilities (e.g. housing, mobility issues, social activities). One community worker argued that filling in a support plan was itself an intervention, as it encouraged older people to articulate needs and reflect on their capabilities. Community workers felt that older people needed guidance in using and strengthening capabilities, taking responsibility for their own health and well-being (e.g. applying for a walker, learning to manage finances). Older people often felt entitled to health and social care services, which they *‘had been working for all their life’* (OP). Community workers thus played important roles in generating awareness of and strengthening older people’s capabilities before turning to (in)formal support, which required careful consideration of when (not) to intervene.

#### Overcoming resistance to informal support

Within the INA, informal support is sought before professional support for older people who cannot meet their own needs. Community workers, however, reported that older people had difficulty relying on informal networks; they were reluctant to ask for help and strongly desired independence: *“People first must drop dead so to speak, before they will turn to help, in other cases they feel you just shouldn’t whine”. (CW)*

Older people especially struggle to ask for social support; despite recognizing its importance, many avoid social contact: *“When they have defined it, it becomes real and that’s so confronting. They got used to being alone and isolated; it became part of their own structure, making them extremely afraid of any change”. (CW)*

Community workers play a crucial role in breaking through this structure and supporting them in seeking contact (Box 1).

Box 1 **real-life case of an INA participant in Rotterdam.**

Mrs. Jansen, a 75-year-old, moved from a big house in a village-like neighborhood to a senior apartment block in an adjacent neighborhood at her children’s encouragement after her husband’s death 6 years previously. Although she initially enjoyed this new home, with nearby shops and well-organized activities, she had lost her sense of belonging and struggled to relate to newcomers: *‘new people are moving in who don’t even bother to say good morning or good evening[…] They pay so much attention to how you walk or how you dress your hair[…] I’m not like that. I’m just an ordinary woman’*. After negative experiences (ridicule at coffee socials, avoidance of invitations to visit), Mrs. Jansen was reluctant to seek social contact, which she missed. She occasionally cried about her husband’s death and longed for someone to talk to. Mrs. Jansen was thus positively surprised when a community worker approached her in the apartment lobby; they sat together and Mrs. Jansen was able to share her story with a neutral person. At Mrs. Jansen’s agreement (and within a week), the community worker arranged for a neighbor to have coffee with her on Monday mornings, which pleases them both: *‘She tells me how happy she is having me over and I also feel very comfortable around her’.* Mrs. Jansen explained that the community worker was essential in setting up this contact. She also appreciated the community worker’s updates about neighborhood activities (e.g. social activities and informational meetings, for example on current reforms in domestic help) and the ability to call someone she trusted whenever she needed support. She was more comfortable opening up to a professional than sharing with one of her hardworking children or neighbors, *‘who have worries of their own’*.

After building relationships and familiarity, community workers are thus crucial in raising older people’s awareness of their (social) needs *and* capabilities, encouraging self-management, and facilitating informal support-giving.

### Micro-level: service integration

#### Engaging community resources

Rather than professional resources, INA community workers utilize locally available community resources and older people’s social networks as much as possible. They engage the community in supporting older people and alerting them to potentially frail individuals. When required services are unavailable, community workers are expected to mobilize volunteers to set up services. In practice, such interventions are not always successful. Two community workers, for example, explained that an informal grocery delivery service they set up at older people’s request remained unused because older people felt it would ‘*threaten their sense of independence*’ (CW) and were anxious about having ‘*an unknown volunteer in their house’* (OP).

#### Service integration: what it takes from professionals

The previous example illustrates that community workers must set up *and* track responses to interventions to support frail older people’s needs. They must play liaison roles at the personal (supporting and monitoring older people), professional (seeking a multidisciplinary approach to support), and community (establishing a well-functioning network and engaging informal support-givers) levels. The INA project manager emphasized the divergence of these tasks: *“Mobilizing the community is completely different from assessing what older people are capable of, which again is different from seeking informal support-givers, without having to throw in a gift card so that they feel valued for what they do. So we expect quite a lot of them”. (PM)*

Participants perceived generalist skills as indispensable, but a health care manager argued that older people may be more inclined to approach community workers based on their specialist, rather than generalist, backgrounds: ‘*I’m not sure whether the ease and trust with which you reach people increases when you position yourself as “being everything” […] I notice that people talk more easily to a caretaker on safety in the neighborhood than their care issues […] The reverse is true as well; it’s easier to talk with a nurse about your prostate disturbances than with a caretaker’* (HCM).

To ensure service integration, community resources must be integrated throughout the process of signaling and supporting older people. Moreover, integrated care and support provision requires community workers to operate simultaneously at multiple levels.

### Meso-level: community integration

#### Building community awareness and trust

Within the INA, community integration relies on community workers’ ability to generate community members’ awareness and trust. Community workers often faced skepticism related to ‘being yet another community project’ and about the INA’s main goals, as utilization of older people’s capabilities and informal networks was often perceived as a way to cut public spending. Moreover, participants wondered whether community members were ready to lose some personal autonomy ‘*in favor of doing something for or with others’* (HCD). Community workers noted that conveying the INA’s message took time and that community members often hesitated to alert them to frail older persons, reluctant to interfere in someone’s life. Community members, for example, shared only ‘justified’ concerns about very frail older people in great need with INA community workers, instead of signaling related to the INA’s target population of those at risk of becoming (more) frail.

#### Familiarity with the neighborhood

Neighborhood-specific familiarity with the preferences of support-givers and those in need of support is crucial for the successful engagement of community members in providing support. One community worker described difficulties in finding a neighbor willing to deliver bread weekly to an older man estranged from society: *‘the whole flat ignored him completely. Although there are quite a few people in that neighborhood supporting others, they seem unwilling to support a person living on the edge of society’* (CW). Neighborhoods may have distinct preferences, standards, and values, which must be considered carefully when providing support to older people: *‘Although Vreewijk is a very cohesive neighborhood, along the way we learned that they uphold the principle of “not washing your dirty laundry in public”, feeling most comfortable in leaving their concerns private’* (PM). Such norms may lead an older person to prefer a support-giver from a different apartment block or street due to fear of gossip. Furthermore, (cultural) differences between neighbors giving and receiving support (e.g. different expectations about support-giving intensity and tasks) may cause problems. One older woman in need of support explained that she knew the match would fail as soon as she saw her potential support-giver walking down the street. INA community workers must take the preferences, and sometimes prejudices, of support-givers and those in need of support into account. Once they find good matches, they notice improvements: *‘People who previously spent their time in their homes now come alive in the neighborhood. There was this isolated man, who now comes to our coffee morning every week*’ (CW).

#### Community integration: what it takes from professionals

The need for community integration requires professionals to reinvent their roles and serve as community workers. A health care organization manager identified this challenge as her greatest concern, wondering whether professionals would successfully attract informal support-givers and perceive collaboration with the community as a self-evident part of their working methods. INA community workers admitted that they struggled to shift from *providing* to *facilitating* support: ‘*I find it very hard and contradictory to gain trust among older people on the one hand, while I should withdraw and facilitate support in the informal network on the other hand’* (CW). Community workers further argued that redirecting older people to professional networks or well-known volunteers was often less time consuming and more reliable than seeking informal support. Although they agreed that neighbors were willing to provide informal support, they emphasized the difficulty of appealing to this sense of willingness. Along the way, they have learned that people’s willingness to support one another is best addressed by articulating concrete, clear requests (e.g. asking whether someone is willing to bring groceries or provide assistance in the garden, rather than whether he/she is willing to do ‘something’ for someone else) and by preventing the excessive formalization of informal support, which would undermine its spontaneous and voluntary character.

#### Sustaining relationships as a prerequisite for community integration

To overcome these barriers to community integration, community workers perceived that sustaining relationships was crucial in gaining access to frail older people and adequately assessing potential support-givers: ‘*it’s about sustaining relationships, that’s why I go to the community center every week, to connect with people, only then do they open up and become willing to collaborate’* (CW). Community workers emphasized that relationships were often person-specific and not easily transferred to other community workers. They thus advocated minimal weekly working hours and project durations to allow professionals to invest in integration among community members and other professionals: ‘*At minimum it requires a year to get a grip on the neighborhood, your own role within INA and the working method of INA. After that you’re able to further refine it’* (CW).

Community integration was thus found to rely on community workers’ ability to gain community members’ trust and the extent to which they became familiar with the neighborhood. Community integration further requires community workers to *facilitate*, rather than *provide*, support.

### Meso-level: professional integration

#### Individual skills

Professional integration starts with selecting appropriate people for the job. Although INA community workers were initially selected for their health and social care backgrounds and familiarity with neighborhoods, the project team learned that entrepreneurial skills were most important. Given the INA’s innovative and complex character, creative community workers who constantly tried new ways to actively reach frail older people and supportive community members most successfully established integration. Community workers’ employment by health and social care organizations in addition to INA work sometimes hampered professional integration. For example, some professionals had to combine INA community work with other functions that necessitated more commercial approaches, which may ‘*lead to a schizophrenic situation in which community workers have to unite a neutral with a commercial attitude; only a few succeed in’* (HET). The question of whether INA community work could best be accomplished by allocating specific tasks–functionalities– to existing professions or by creating new, specific INA professions was a recurrent theme during meetings. Most partners agreed that community workers should combine a generalist scope with specialist backgrounds, enabling determination of when (another) specialty is required to support an older person and ensuring high-quality person-centered support.

#### Team skills

To facilitate professional integration, community teams must incorporate various specialties, ‘*combining their skills to ensure a generalist and holistic approach’* (PM). The availability of an appropriate range of skills and expertise on a team was perceived as a prerequisite for professional integration, and particularly relevant for the INA’s focus on improving *overall* well-being. One community worker, for example, commented: ‘*I brought my knowledge about health care to the table and how to approach older people […] And I taught the social worker how to cope with older peoples’ sexual impulses[…] and the other community worker had great entrepreneurial energy, which I found very stimulating”* (CW). Membership in a diverse community team seemed to generate more than the sum of its parts: *‘soon I started to feel that we could conquer the world[…]you learn to recognize symptoms that up until then weren’t natural for me’* (CW).

Community workers, however, emphasized that team synergy could be achieved only when team members were receptive to professionals from other disciplines and were able to address relational issues that may hamper the establishment of mutual goals. Continuity within the team was thus perceived as a prerequisite for professional integration. One community worker explained that changes in team composition harmed collaboration: *‘Every time we needed to start from scratch, how do we communicate, what are our intentions?’* (CW). Moreover, community workers perceived the imposition of output criteria and targets as the greatest threat to collaboration: ‘*If one of us generates a lot of clients, and the others don’t, it sure causes friction’* (CW). Community workers expressed concern about meeting the target of identifying frail older people: ‘*Although we planned to go to a senior apartment block together, one of the community workers went there before; it sure makes you doubt whether there are any elderly people left for you’* (CW). The establishment of team, rather than individual, targets may overcome this barrier.

Recruitment of ‘entrepreneurial’ professionals with generalist and specialist skills to form diverse teams was thus found to be crucial for professional integration in the support of older people with varying and complex needs. Although teams may generate more than the sum of their parts, discontinuity and a lack of mutual goals were found to hamper professional integration.

### Meso-level: organizational integration

#### Conflicting organizational interests

Although health and social care organizations recognize the need to collaborate, professionals feel that cost containments are forcing the prioritization of organizations’ interests over the common good: ‘*in times of reforming a structure, in times of insecurity concerning the survival of organizations, you have to save your own skin, and that’s when the power of the institute becomes way too large in the procedure’* (HCD). Although INA directors and managers displayed a lack of confidence in achieving organizational integration on an institutional level, due to their need to meet organizational targets in order to ‘survive’, they *did* feel that collaboration succeeds on an operational level on the basis of mutual understanding and acknowledgement. Although they seemed confident, community workers constantly noted that competition among professionals hampered organizational integration. In addition to expressing the general fear of failing to meet their targets, professionals identified the ‘blurring’ of professional identities, i.e. lack of clear roles, as an important impediment to organizational integration; one community worker commented: ‘*I still find it very strange that community nurses [community workers with similar but more health care*–*related tasks] are getting all these extra tasks. They may say the former community nurse did the same, but then they’re talking about the 50s, when the milkman put the bottles on the curb; it’s a completely different and complicated world now’* (CW). Ill-defined roles not only led to confusion among older people and professionals, it also encouraged INA community workers to constantly explain and justify their roles, even within their own organizations. This sense of competition hampered INA community workers’ provision of support to older people; one community worker explained that she was opposed by an activity coordinator when she tried to organize activities in a flat that was crowded with isolated older people: *‘We had put an INA folder in the mailbox, which made the activity supervisor very irritated. She argued that they didn’t need it and that they had their own activities[…]but it’s a three-by-three apartment with no balcony, it’s like a prison’* (CW).

#### Lack of organizational commitment

Community workers were equally disappointed in their organizations’ and managers’ lack of engagement during the project. They were seldom asked about their INA experiences, and meetings called by management merely involved elaboration on practical issues, such as sick leave or investment of time in the INA. One community worker felt appreciated only *‘for delivering clients through the project’*, whereas she had hoped her INA experience would foster innovation in her organization. Similarly, the INA project manager stated that he had placed too much trust in health and social care organizations’ commitment. Furthermore, he identified a lack of structural incentives that would generate organizational integration; during an advisory group meeting within the INA context, *‘they kept going on about who was responsible for which domain and about the sense of competition or collaboration among health and social care […] And suddenly it struck me that there was no other meeting where they encountered each other’* (PM). Thus, successful partnerships often involved willing professionals or managers or depended on high levels of trust built through previous collaboration. This specifically accounted for the INA engagement only of general practitioners who felt affiliated with the need to support community-dwelling older people. Managers’ active interference was felt to promote organizational integration. For example, when INA community workers indicated that community nurses from a similar but more health care–oriented program perceived them as valuable only when no other way to support an older person had been found, both program managers held a meeting to integrate services provided by community workers and nurses. The managers’ expression of mutual commitment to collaboration, through organization of this meeting and on-site articulation of their engagement, made community workers perceive collaboration as an indispensable part of their job. Moreover, regular discussion of clients or provision of feedback to professionals who had identified potentially frail older people to INA workers enabled professionals to see their complementary values.

Within the INA, organizational integration was thus impeded by conflicting organizational interests and achieved only under favorable conditions, i.e. through a few willing professionals or managers and through high levels of trust built during previous collaborations. Structural incentives, such as the creation of opportunities for professionals to meet and gain insight in each other’s added value, facilitate organizational integration.

### Macro-level: system integration

#### Inadequate financial incentives

Participants identified divergent flows of funds as the main cause for the lack of adequate financial incentives, affecting health and social care organizations and municipalities: *‘When the municipality performs its tasks regarding the Social Support Act [The Social Support Act took effect in 2007 and requires municipalities to meet increasing legal responsibilities regarding support of people (with disabilities)] well and arranges prevention properly, it won’t benefit the municipality; it will only lead to lower expenditures among health insurers. Well, do you think that’s of any interest to the average civil servant?’* (HI).

The health insurer and municipal officers argued that incentives should ensure that incremental improvements bring economic benefits for all stakeholders, facilitating work toward the same goal, i.e. integrated care and support provision to older people. Current financial systems lack stimuli for innovation; the health insurer commented: ‘*I’d also prefer to build in a reward for innovative behavior. And that’s very complicated, what you basically see is that those who act last in this transition, or focus on its production, win this race financially’* (HI). During meetings held to discuss whether and how the INA could be sustained after project funding ended, partners looked to each other with the hope of (financial) commitment. Participants emphasized the need for broader (financial) commitment to sustain approaches such as the INA: *‘On the content, we agree with each other, but there are other sides to consider as well. The question is whether we can commit ourselves jointly […] if you ask an individual organization if they’re willing to commit, while having to cut back intensely… it demands a broader approach in which all parties commit’* (HCD).

#### Inadequate accountability incentives

Similarly, health and social care organizations urged the municipality to reconsider its accountability incentives, annoyed by the focus on *how* they do things: *‘They use accountable performance indicators such as the amount of hours spent… because that’s the most measurable aspect […] When I’m talking with a municipal officer, 90% of the conversation turns to talking about a spreadsheet with the amount of hours of our employees’* (SCD). A sub-alderman argued that this focus compromised municipalities’ interests, namely the need to innovate and empower citizens to participate: ‘*we currently steer on “fifteen minutes of this and fifteen minutes of that […] and it should also meet these and these conditions”; so we’re very much on the details of how to do it. But when the job is to attract as many volunteers to empower social networks, then you should provide them the necessary space to do so’* (SUBALD). Instead of focusing on process, creating a bureaucratic accountability system, many participants would prefer the municipality to promote results and focus on result-oriented indicators.

#### Inadequate regulatory incentives

Paradoxically, professionals experienced similar restrictions. Community workers are told that the provision of high-quality support requires innovation and collaboration among community partners while being required to bureaucratically account for all actions and meet targets. A health care director and a health insurer expressed the wish that professionals would seek ways around these constraints, taking the system and its operational rules rather lightly. The health care director explained how he would like two community nurses from different organizations to collaborate in the community: ‘*I actually hope they’re being smart about it, like “you know what, I’ll give you a hand, or I’ll do it for you this time”. Without having to send an invoice on either side’* (HCD). However, operational rules seem to restrain professionals’ autonomy on such occasions.

On a macro-level, the INA was affected by the system’s failure to provide adequate financial, regulatory, and accountability incentives. Current system incentives lack a clear division of tasks and fail to generate broad engagement. To enable successful integrated care and support efforts, incentives should carefully anticipate the needs for innovation and collaboration. This approach requires financial incentives that account for aligned incremental improvements and accountability measures that provide professional autonomy.

### Functional integration throughout all levels

#### The risk of excessive professional autonomy

The INA’s innovative character, specifically with respect to active community engagement, created a paradoxical situation. The project leader gave community workers autonomy to create their own working methods, with no guideline or restriction on how they spent their hours. This autonomy was a main motivation to become an INA community worker, as professionals missed it in their regular jobs. However, joint training conducted 1.5 years after INA initiation revealed a discrepancy between community workers’ and project management’s perspectives on core tasks. The trainer concluded that community workers did not yet perceive community engagement and a facilitating role as self-evident parts of their job. She stated that community workers remained ‘*bound by the conventional way of organizing things, i.e. from the perspective of helping/fixing problems’* (HET).

#### Lack of support tools

The lack of clear interventions or decision support tools paralyzed community workers, forcing them to rely on usual working methods. They were expected to develop support plans, but the process had not been fine-tuned for everyday practice. Given the lack of tools and guidelines to support community workers’ decision making, the plans became a formality instead of a supportive tool. Team meetings neither were a resource for aligning professional standards or gaining confidence in the value of the work. Whereas most community workers needed to describe their struggles and discuss community issues, meetings were focused predominantly on practical issues (e.g. whether targets were being met): *‘I have a strong need to get inspired and informed. I wonder why we don’t discuss such things, I read stuff in the local newspaper which I think we should address and which is being addressed by residents in the community centers’* (CW). Discussion of local and broader (e.g. transitions in municipal and central government) issues would also contribute to workers’ understanding of the context in which they operated, fostering their sense of purpose. Encouraged by INA’s education team, the project manager decided to include discussions of successful cases/situations and those with which community workers struggled in team meetings. For example, one community worker was reassured that she was allowed to spend 2 hours on a bench in front of the supermarket if it facilitated her acquaintance with the neighborhood and its (older) inhabitants.

#### High touch, low tech

In exchanging information, community workers often applied a ‘high touch, low tech’ approach. Rather than using the web-based portal developed for the INA, community workers preferred to consult each other by telephone or in person. These ‘*short lines of communication*’ (CW) were considered to be most valuable for team collaboration. One community worker, however, expressed her preference for a handheld tablet to assist with fieldwork: ‘*I can’t bring all my paperwork on the street. Give me an iPad and I can access all the information: which volunteer is available, for example. I’m racking my brains out there’* (CW).

Professional autonomy provided by project management was at odds with guidance in adopting a new professional role that matched the INA’s core principles. The INA’s innovative character increased community workers’ need for guidance and supportive tools. The lack of material (i.e. decision-support tools or guidelines) and immaterial (i.e. acknowledgement) resources hampered the creation of shared values and aligned professional standards.

### Normative integration throughout all levels

The dynamic environment in which the INA operated seemed to overshadow the urgency to facilitate an integrated mind-set. Rotterdam’s use of competitive tender practices to appoint (new) providers and award contracts impacted INA organizations and community workers. Although these practices and other policy changes (mainly in home care) aimed to increase the efficiency of integrated care and support provision, they created marked insecurity, impeding the INA’s ability to generate multilevel integration.

#### Insecurity and mistrust

For older people, tender practices and policy changes often implied the rationing of publicly funded health and social care services and discontinuity in service delivery. Older people thus have become insecure and feel that they are burdening society: ‘*in roughly one and a half years they restructured all home care services… And they may argue that volunteers will cover those things that remain to be done, but we must wait to see who’s coming […] It feels like we don’t matter anymore’* (OP). The INA’s anticipation of these transformations by shifting responsibilities back to the community frightened older people and confirmed the idea that the INA was *‘no more than a hidden economic measure’* (OP). Furthermore, based on previous experiences, older people associated the INA with a negative form of social control: ‘*The problem is that those of the younger generation are not familiar with a cohesive community and I think that those from the older generation who still remember that world can´t relate to it anymore’* (HCD).

Tender practices also generated mistrust among health and social care professionals. Many professionals commented that they did not understand *‘why the municipality first imposed major cutbacks’* (CW), leading to community center closures and job losses among very experienced community workers, and then forced them to rebuild services. These practices drew energy away from the support of older people through the INA. The project manager argued that this situation paralyzed community workers and prevented the INA from making a real transition: *‘It caused a standstill. The community workers were caught by insecurity and passivity for at least half a year. There was only room for bereavement’* (PM). The INA’s expectations concerning deprofessionalization further increased professionals’ mistrust, causing a conflict of loyalty toward the INA.

Municipalities were similarly affected by a high degree of insecurity: ‘*Until January 2015 we won’t know how much money we’ll get from the state[…]But what’s even more fundamental, is that the Bill of the Social Support Act won’t be ready until mid-2014, and that should provide us with the instructions and the conditions under which we must operate. But by that time our procurement should have long been realized. So that’s a very strange situation’* (MO). In an interview conducted 2 days before his resignation, a municipal officer described the resulting risk-averse culture within municipalities, which prohibited ‘*thinking out of the box and trying innovative approaches’* (MO). Although municipalities shift responsibilities to (social) care organizations and communities, they concurrently try to retain top-down control; the same municipal officer commented: *‘we supposedly have marketed it, but on the other hand, we still held on to legislation, which makes no sense’* (MO). Paradoxically, municipalities’ constant tendency to control and prevent risks so that frail people don’t ‘fall through the cracks’ causes mutual distrust, undermining collaboration and innovation; a municipal program manager commented: ‘*We face very complex strategic decisions, and of course there is no mutual trust. It seems very simple, but trust in one another is a key driver in this sector; are we actually supporting people who are in need or are we just earning money on their backs?’* (MO). The widespread culture of accountability thus causes organizations to focus on their own interests instead of committing to an integrated mind-set that focuses on the best interests of (frail) citizens.

## Discussion

This study showed that integrated care and support provision through an INA is a complex, dynamic process requiring multilevel alignment of activities [18]. The INA achieved integration at the personal, service, and professional levels only occasionally. Micro-level bottom-up initiatives were not aligned with top-down incentives, forcing community workers to establish integration *despite* rather than *because of* meso- and macro-level contexts. Functional and normative integration were lacking, with excessive reliance on professionals to achieve integration.

Incoherent macro-level policies have been identified as main barriers to the pursuit of integration. Current system incentives are not aligned to achieve collaboration and innovation and do not account for the complexity and nature of issues arising locally. In line with previous findings [11], health and social care partners identified divergent flows of funds and the lack of joint budgets as significant obstacles to collaboration. Current performance indicators prioritize accountability and control, rather than creating a learning environment that allows partners to try innovative approaches [25]. Thus, health and social care partners advocate that the government is ‘tight on ends and loose on means’. However, municipal officers and health insurers expressed concern that allowing local variations in means may cause (frail older) people (to whom they are legally required to provide support) to ‘fall through the cracks’.

This tendency to control and prevent risks while being in need of innovation and collaboration affected the professional and organizational levels. Although managers and directors were confident that professionals would seek ways around system constraints, our research demonstrates that professional and organizational collaboration requires appropriate structural incentives. The creation of opportunities for professionals and managers to meet and gain insight into their complementary roles is crucial. Without an aligned macro-level policy narrative, bottom-up initiatives such as the INA will struggle to make impacts.

Overcoming these macro-level barriers is necessary – but not sufficient – for integration [18,21,26,27]. The lack of normative integration fundamentally prevented the INA’s integration of care and support. The rate and complexity of current reforms were detrimental to established community relationships and generated high levels of mutual distrust and insecurity throughout system levels. Professionals and organizations re-focused energy on individual interests [26,28,29], rather than working toward the common goal of improving care and support for older people. In line with previous findings, such dynamic environments hampered the development of an innovative culture [30].

To promote normative integration, trust may be more determinant than streamlined structures [31]. Trust was a recurrent theme at the personal, community (as a prerequisite for older people’s and community members’ commitment) and professional (as a pre-existing factor built through previous collaboration that enabled professional integration) levels. These findings emphasize the importance of continuous relationships that allow the development of trust and social capital in pursuing integration [1,31,32]. Restructuring efforts may cause ‘cultural damage’ by undermining the importance of trust and relationships for normative integration [29].

Our study also revealed a lack of functional integration. Material and immaterial support tools were insufficient for the creation of shared values and aligned professional standards. Although a protocol-driven approach would conflict with the need to provide tailored care to older people with complex needs, the INA’s innovative character increased the need to support change and direct professionals toward mutually agreed-upon objectives and practices [11]. Support tools must be responsive to professionals’ struggles and the need for innovation while respecting professional autonomy and diversity.

Although not addressed in many integrated care and support models, the community level was found to be critical in engaging community members and resources when meeting older people’s needs. Our study indicated the importance of community workers’ understanding of community standards and norms. Furthermore, professionals struggled to perceive community members’ roles as integral to the support-giving process [33]; guidance of professionals in engaging informal support-givers is thus crucial in promoting community integration. Our study revealed clear barriers to informal support, suggesting that its provision and receipt require a paradigm shift toward more natural occurrence and self-evidence.

Although this study provides knowledge about factors that promote or hinder integration at the micro-, meso- and macro-levels, the context-specific nature limits the generalizability of its findings. However, we feel that our detailed and multi-faceted description of diverse INA partners’ experiences provides useful insights for future research. The INA took place in a highly dynamic environment with intense external forces, which impacted the success of integrated care and support provision. Further research should account for interactions between external factors and local integrated care and support delivery processes from all perspectives including the perspectives of older people. Successful integration within a complex program such as the INA requires time, continuity, and broad commitment throughout levels, with evolution toward aligned norms and practices. Moreover, we demonstrate that the community level should be included in integrated care and support models, as specific (social) community characteristics must be considered when improving community-based integrated care and support. Future research should also focus on the development of validated measurement tools to assess the ‘strength’ of integration throughout levels and its impact on (cost) effectiveness.

## Conclusions

This study enabled us to identify factors facilitating and inhibiting integration within and among levels defined by Valentijn and colleagues [19]. This integrated care model enabled us to acquire a rich understanding of the INA’s underlying processes, which most integrated care and support initiatives fail to do. Although this model, like most integrated care models, focuses predominantly on the improvement of *health* outcomes, instead of aiming to improve overall well-being, it was useful for the detection of contextual factors and mechanisms that may hinder or facilitate an INA. However, our findings highlighted the need for further refinement of the model by adding the community level [33]. This level often is not ‘incorporated in our theorising on integrated care’, as Nies [8, p. 3] and Goodwin [9] recently remarked. Our study indicated that this community level is indispensable in engaging community members and resources to meet older people’s needs. Given the general shift in the primary provision of (social) care from the state to the community, community engagement is increasingly essential. Our research identified several barriers to the pursuit of community integration. To overcome these barriers, neighborhood-specific familiarity with the preferences of support-givers and those in need of support may be crucial for the successful engagement of the community. Our study also enhanced our understanding of the importance of *normative* integration in INA development. Relational and normative aspects may be best accounted for in what Goodwin [9, p. 2] describes as a *culturally sensitive approach*; an approach that aims to build community awareness and trust among formal and informal partners. Through our multifaceted and thorough description of the experiences of diverse INA partners, we were able to test Valentijn and colleagues’ (2013) integrated care model and provide a richer account of its implications. These findings are especially important in a time of ageing populations and a general shift in the primary provision of (social) care from the state to the community.

## Competing Interests

The authors declare that they have no competing interests.
